# SNP array and FISH analysis of a proband with a 22q13.2- 22qter duplication shed light on the molecular origin of the rearrangement

**DOI:** 10.1186/s12881-015-0193-y

**Published:** 2015-07-07

**Authors:** Chiara Magri, Eleonora Marchina, Valeria Bertini, Michele Traversa, Giulia Savio, Alba Pilotta, Giovanna Piovani

**Affiliations:** Biology and Genetics Division, Department of Molecular and Translational Medicine, University of Brescia, Viale Europa 11, 25123 Brescia, Italy; Pediatric Division, Spedali Civili, Brescia, Piazzale Spedali Civili 1, 25123 Brescia, Italy

**Keywords:** SNP-array, CNV, Translocation, Duplication, 22q13.2, 22qter, BIR

## Abstract

**Background:**

In about one third of healthy subjects, the microscopic analysis of chromosomes reveals heteromorphisms with no clinical implications: for example changes in size of the short arm of acrocentric chromosomes. In patients with a pathological phenotype, however, a large acrocentric short arm can mask a genomic imbalance and should be investigated in more detail. We report the first case of a chromosome 22 with a large acrocentric short arm masking a partial trisomy of the distal long arm, characterized by SNP array. We suggest a possible molecular mechanism underlying the rearrangement.

**Case presentation:**

We report the case of a 15-year-old dysmorphic girl with low grade psychomotor retardation characterized by a karyotype with a large acrocentric short arm of one chromosome 22.

Cytogenetic analysis revealed a normal karyotype with a very intense Q-fluorescent and large satellite on the chromosome 22 short arm. Fluorescence in situ hybridisation analysis showed a *de novo* partial trisomy of the 22q13.2-qter chromosome region attached to the short arm of chromosome 22. SNP-array analysis showed that the duplication was 8.5 Mb long and originated from the paternal chromosome. Haplotype analysis revealed that the two paternal copies of the distal part of chromosome 22 have the same haplotype and, therefore, both originated from the same paternal chromosome 22. A possible molecular mechanism that could explain this scenario is a break-induced replication (BIR) which is involved in non-reciprocal translocation events.

**Conclusion:**

The combined use of FISH and SNP arrays was crucial for a better understanding of the molecular mechanism underlying this rearrangement. This strategy could be applied for a better understanding of the molecular mechanisms underlying cryptic chromosomal rearrangements.

## Background

In about one third of healthy subjects, the cytogenetic analysis of chromosomes reveals heteromorphisms with no clinical implications: for example changes in size of the short arm of acrocentric chromosomes These regions include three cytogenetic bands in which there are several types of tandem repeated DNA sequences: satellites I, II, III, IV, distal beta-satellite, multiple copies of rRNA genes, proximal beta satellite DNA, and telomere sequences [1–3].

A change in size of the short arm of an acrocentric chromosome is considered as a benign variant, however, the detection of a variant chromosome, in a subject with an abnormal phenotype, should be investigated in more detail using cytogenetic and molecular technologies. Ravnan et al., [4] found a group of unbalanced translocations, involving a sub-telomere region of an acrocentric chromosome, and showed that apparent acrocentric short arm polymorphisms can indeed represent significant genomic imbalances.

In this report, we describe the combined use of fluorescence in situ hybridisation (FISH) and single nucleotide polymorphism (SNP) array technologies to study the case of a large acrocentric short arm of a chromosome 22 which masks a *de novo* trisomy of a distal portion of the long arm of chromosome 22 in a 15-year-old dysmorphic girl with mild psychomotor retardation. Only 19 cases of pure partial trisomy involving the distal long arm of chromosome 22 have been reported [5–19] three of which have been characterized using a CGH-array [13, 14, 19]. We believe that the case reported in this paper is the first analysed with SNP array. In most of the pure trisomy of distal 22q, duplication is the consequence of a *de novo* non-reciprocal translocation on the short arm of an acrocentric chromosome or a recombination event of a pericentric inversion in the parent. However, the molecular mechanisms underlying this chromosome rearrangement remain unclear [9].

We demonstrate how the combined use of FISH and SNP genotype array information may be useful to determine the parental origin of the duplicated portion of chromosome 22 and to hypothesize a possible molecular mechanism of formation.

## Case presentation

### Clinical Report

The proband was the second of two sisters born from healthy, non-consanguineous, Caucasian parents. At the time of birth, the mother was 32 and the father was 28 years old; the sister is healthy and familiar history was negative for malformation or developmental delay. The patient was born at 42 weeks by caesarean section because of fetal bradycardia; maternal gestational diabetes was evident from the fifth month of pregnancy, no prenatal diagnosis was made. The birth weight was 3.2 kg (25–50 percentile), length was 49 cm (25 percentile), and the Apgar score was 8/10. The neonatal period was unremarkable, congenital hip dysplasia was immediately evident and specific support was applied for three months.

At five years of age, when starting school, a delay in psychomotor skills and language was present, cognitive performance was evaluated by the Wechsler Intelligence Scale for Children (WISCH), together with writing and reading tests. Low-grade mental retardation was evidenced. Psycho-pedagogical help was provided and the primary school was successfully completed. The growth was normal in terms of height, 25 percentile (Tanner scale), and weight however, until 11 years old, the bone age was delayed by two years compared to the chronological age, from 12 years, both ages were similar. Puberal development was at 12.5 years and menstrual cycles were regular.

At the last examination, the 14.9 year-old patient attended a secondary technical school, her height was 157.8 cm (25 percentile), weight was 44.2 kg (3 percentile), the head circumference was 50.5 cm (<3 percentile) and sexual development had been completed.

**Fig. 1:**
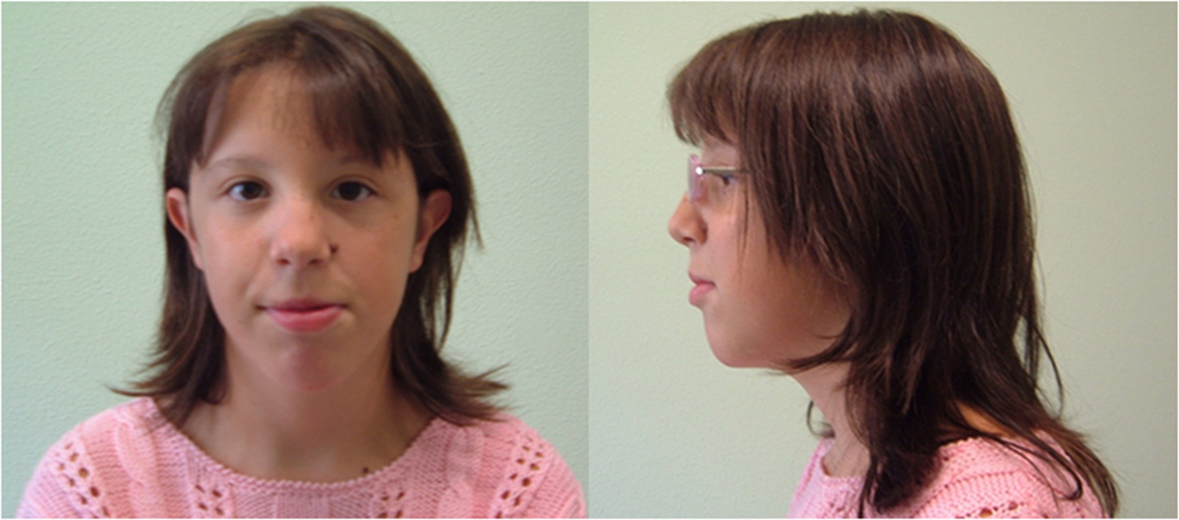
Photographs of the patient at the age of 15. Frontal and lateral view of the proband showing mild dysmorphic traits

Physical examination showed some dysmorphic features: microcephaly, long face, sparse eyebrows, telecanthus, low set and laterally protruding ears, long philtrum, macrostomia, prominent lower lip, large ear, gibbus deformity, bilateral clinodactyly of the fifth finger of hand (Fig. 1). No psychiatric signs were observed. Ultrasound examination of heart and other internal organs gave normal results.

## Materials and methods

Chromosome preparations were obtained with phytohaemoagglutinin stimulated lymphocytes from a peripheral blood sample. Cultures were set up and harvested by standard methods. Cytogenetic analysis was performed using Q and G-banding at 450 bands resolution and using C, Nucleolus Organiser Region (NOR) and Distamycin A and 4′,6-diamidino-2-phenylindole (DA-DAPI) stainings according to the International System for Human Cytogenetic Nomenclature [20, 21]. The Chromoprobe Multiprobe-T System (Cytocell Ltd) was used to screen the subtelomeric regions according to the manufacturer’s protocol.

FISH analysis was carried out by standard procedures as described previously [22]. Dual-colour FISH experiments were performed with Bacterial Artificial Chromosome (BAC) clones reported in Table 1 and provided by Chori (BAC PAC Resources Center Children’s Hospital Oakland Research Institute in Oakland, California, USA). 4′,6-Diamidino-2-Phenylindole, Dilactate (DAPI) staining was used for chromosome identification during FISH analysis. FISH signals were analysed using a Zeiss Axiophot microscope with a triple bandpass filter to simultaneously detect signals. Images were collected and merged using a cooled CCD camera.

**Table 1:** BAC clones used for dual colour FISH experiments

BAC clones	Position on Chr22 (Hg19)	Dual colour FISH		Notes
		1st FISH	2nd FISH	
RP11-241G19	42,605,118-42,782,007	green		Located outside the duplicated region
RP11-140I15	46,097,912-46,268,798		green	
RP11-164E23	50,841,120-50,995,394	red	red	The most telomeric RP11 probe available on Chr22q.

The DNA of the proband and of her healthy parents were analysed with GeneChip Human Mapping 250 K *Nsp*I arrays (Affymetrix). The DNA was processed following instructions provided in the Affymetrix GeneChip Human Mapping 500 K Assay Manual. Initial analysis and quality assessment of the array data were performed using GTYPE 4.1 (Affymetrix). Copy number state determination was performed using CNAT4 software (Affymetrix) by comparing our samples with a reference set of 48 Hapmap samples available on the Affymetrix web site (http://www.affymetrix.com/support/technical/sample_data/500k_data.affx).

**Fig. 2:**
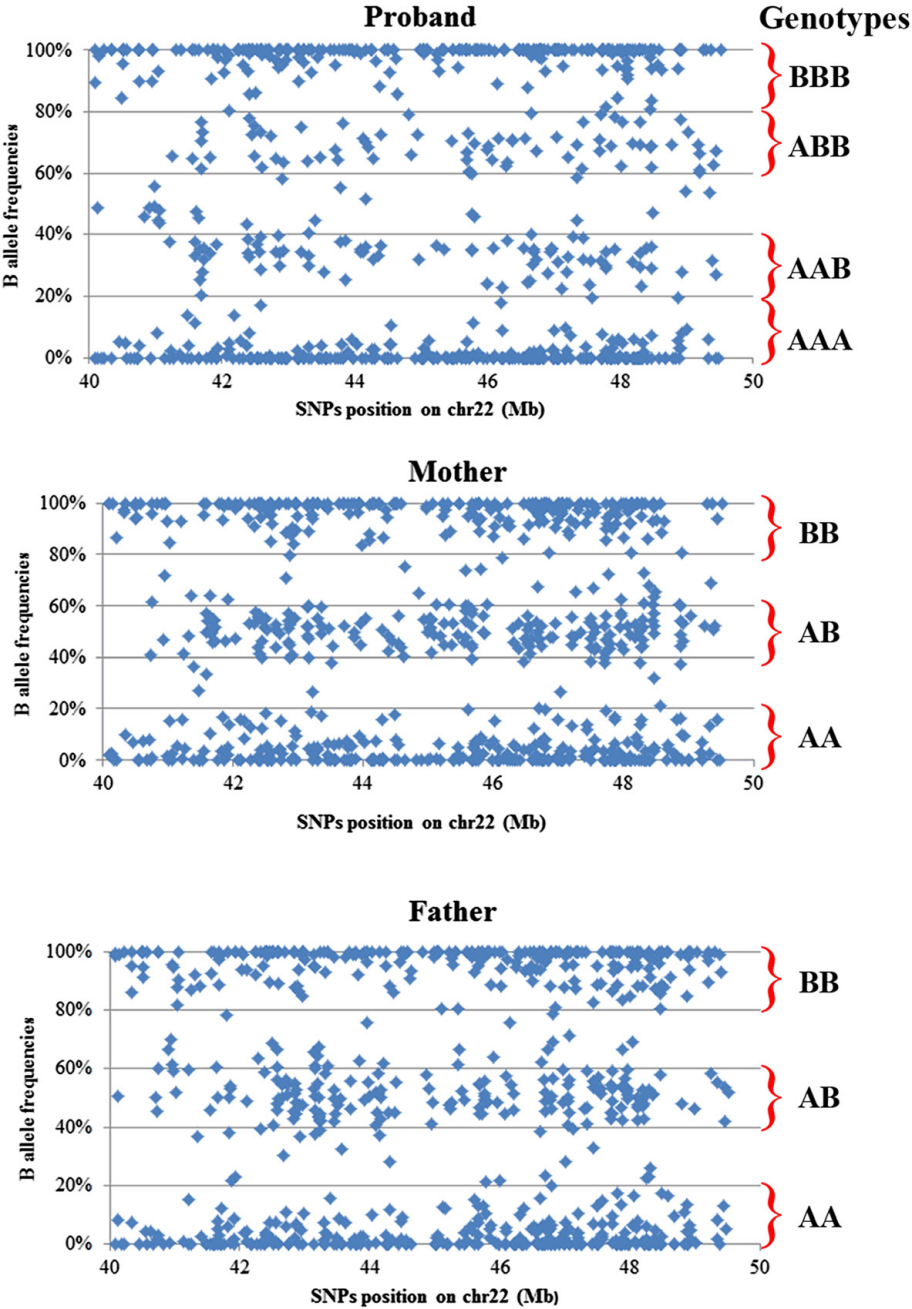
Scatterplots of the relative B allele intensity signals. On the right, B allele intensity signals for the SNPs in the duplicated portion of chromosome 22 in the proband and her parents, respectively. On the left, the genotype assigned to each probe according to their B allele frequencies

Concerning the duplicated region, Affymetrix Power Tool software was used to extract “allele specific” signal values from the raw CEL file for those SNPs falling in the duplicated region. “Allele specific” refers to the fact that for each SNP, there is a signal measure for the A allele and a separate signal measure for the B allele. The relative intensity signal of the B allele was then used to assign the genotype. The homozygous BB genotype was assigned to B intensity values between 1 and 0.8, ABB to values between 0.8-0.6, AB to values between 0.6-0.4, AAB to values between 0.4-0.2 and AA to values between 0.2-0.0 (Fig. 2).

The bioinformatics analysis of the sequences involved in the chromosomal rearrangement was carried out using the University of California Santa Cruz (UCSC) Genome Browser (http://genome.ucsc.edu/) on the GrCh37 Feb 2009 release of the human genome.

## Results

**Fig. 3:**
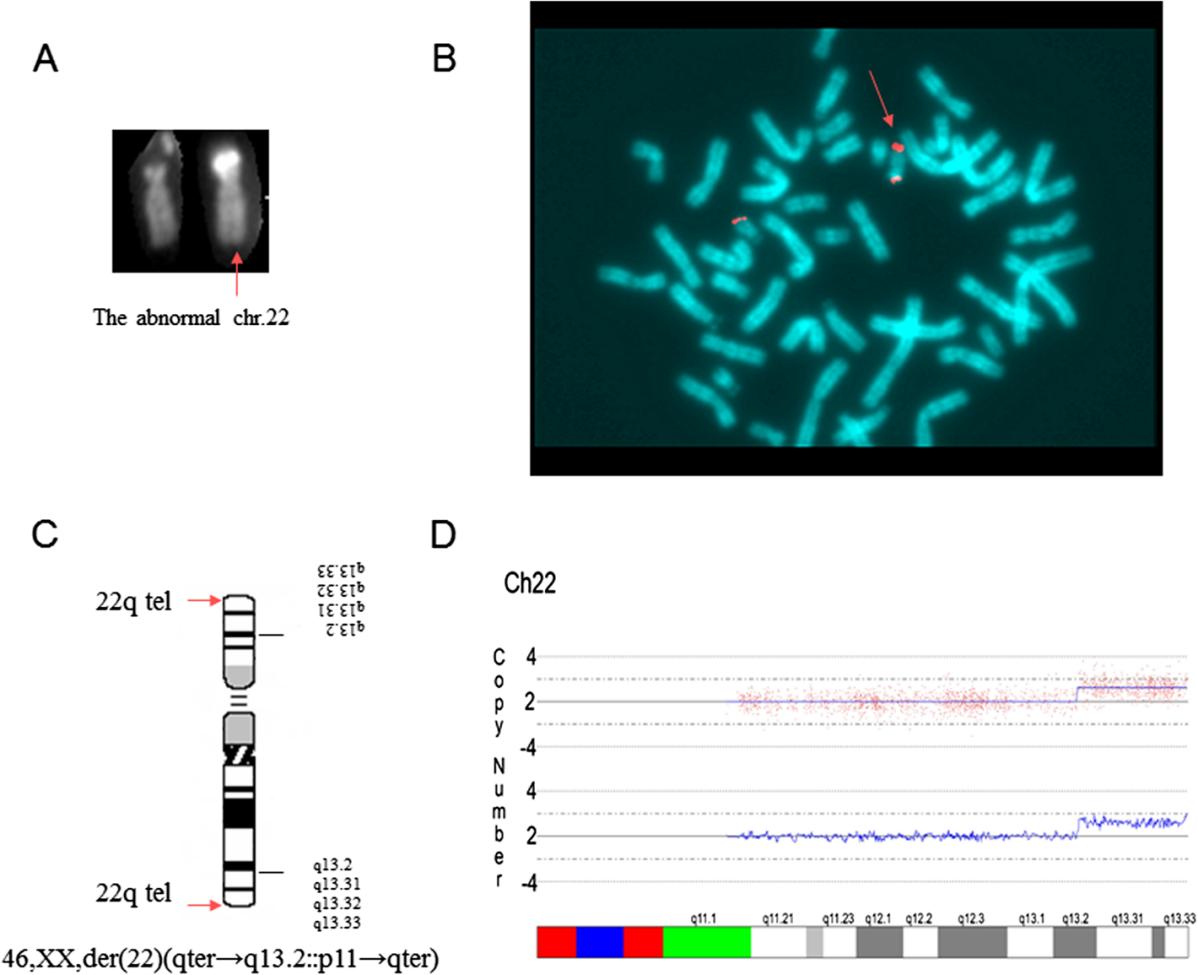
Cytogenetic and molecular analyses. **a** chromosome 22 QFQ banded karyotype of the patient. The red arrow points to the abnormal chromosome 22. **b** FISH analysis with probes for the subtelomeric region of chromosome 22q (in red). An additional signal located on the short arm of chromosome 22. **c** The derivative ideogram of the chromosome 22q13.2-qter duplication; **d** Array analysis. Copy number state of chromosome 22 probes inferred by CNAT and reported as number of copies

Cytogenetic analysis of the proband revealed a normal karyotype, however Q banded analysis showed the presence of one normal chromosome 22 and one submetacentric chromosome 22 with a very intense Q-fluorescent and large satellite on the short arm (Fig. 3a). C banding confirmed the presence of only one centromere, staining with silver nitrate revealed the presence of a NOR in the p arm, while DA-DAPI staining was negative.

**Fig. 4:**
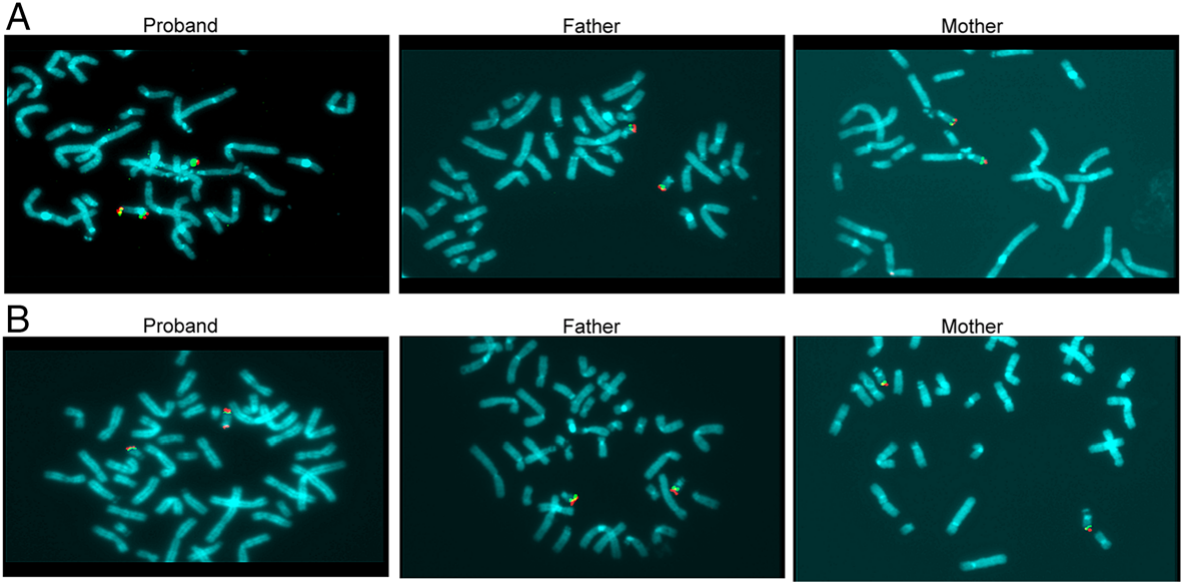
Dual Colour FISH analyses. **a** FISH analysis with probes RP11-140I15 (green) and RP11-164E23 (red). In the proband, two additional hybridisation signals on the abnormal short arm of chromosome 22 are visible, whereas in the parents only two signals in the correct orientation are visible on the long arm of chr22. **b** FISH analysis with probes RP11-241G19 (green) and RP11-164E23 (red). The RP11-241G19 probe is located outside the duplicated region and displays only one hybridisation signal on the long arm of chr22 in all the subjects

FISH with telomere set probes revealed an additional positive hybridisation signal of the specific subtelomere probe of the 22 chromosome long arm on the large satellite of the short arm of chromosome 22 (Fig. 3b). To rule out the presence of cryptic imbalances in the genome and to confirm our results, we performed further investigations using BAC clones (RP-11) specific for different regions of chromosome 22q13.2 to characterize the duplicated region. Dual-colour FISH with BAC clones RP11-140I15 and RP11-164E23 showed two hybridisation signals on the abnormal short arm of chromosome 22, whereas dual-colour FISH with BAC clones RP11-241G19 and RP11-164E23 revealed only one hybridisation signal of BAC RP11-164E23 on the abnormal short arm of chromosome 22. Based on these findings, we determined the karyotype to be: 46,XX, ish der(22)(qter → q13.2::p11 → qter) [21], (Fig. 3c). FISH with telomeric and with the same BAC clones revealed normal results in the parents: no rearranged or inverted cryptic duplicated regions were highlighted (Fig. 4).

The extension of the duplication was characterized by SNP array. The analysis with Affymetrix GeneChip Human Mapping 250 K array showed that the duplication was 8.5 Mb long and that the starting point mapped between 42806124 bp (SNP_A-1904331, first non-duplicated probe) and 42875479 bp (SNP_A-4229298 first duplicated probe) (Fig. 3d). The SNP array analysis of the parents’ DNA confirmed the *de novo* origin of the duplication. To verify the paternal or maternal origin of the duplication, the Mendelian segregation of 137 probes, for which the proband was heterozygote (AAB or ABB), were analysed (Table 2). Eleven probes were not informative since both parents were heterozygotes. For 53 SNPs (39 %), the duplicated allele could have been inherited from either the mother or the father. None of the duplicated alleles were clearly inherited from the mother, whereas for 73 SNPs (53 %), the duplicated allele was undoubtedly of paternal origin (Table 2). These data demonstrate that the duplicated portion of chromosome 22 arose on the paternal homologous.

**Table 2:** Allele transmission pattern for proband heterozygous SNPs in the 22q duplicated region

Proband heterozygous genotypes	Mother genotype	Father genotype	Parent transmitting two alleles (alleles)	Tot. SNPs
AAB	AB	AA	Mat(AB) or Pat(AA)	32
	AB	AB	Not informative	5
	BB	AA	Pat(AA)	13
	BB	AB	Pat(AA)	21
ABB	AA	AB	Pat(BB)	17
	AA	BB	Pat(BB)	22
	AB	AB	Not informative	6
	AB	BB	Mat(AB) or Pat(BB)	20
	BB	AB	Mat(BB) or Pat(AB)	1
Total SNPs				137

Interestingly, the father transmitted always two identical alleles (AA or BB) to his daughter (Table 2). Only one probe circumvented this rule, likely related to a genotyping error in one of three samples. This demonstrates that the two paternal copies of the distal part of chromosome 22 have the same haplotype and, therefore, both originated from the same paternal chromosome 22.

## Discussion

Pure duplication of the distal long arm of chromosome 22 (22qter) seems to be extremely rare, in fact only 19 cases of pure trisomy 22qter have been described to date [5–19]. Fenstra et al., [9] subdivided pure trisomies 22qter into four groups according to the size of the duplicated region: large (22q12-qter), intermediate (22q13.1-qter), small (22q13.2-qter) and smallest (22q13.3-qter) duplications. Usually the severity of the clinical phenotype correlates with the dimension of the duplicated region. However, with the exception of patients with the smallest duplications, who have a consistent clinical presentation leading to moderate/mild mental retardation, microcephaly and similar mild dysmorphic traits [14], patients with apparently the same duplicated region show a wide spectrum of phenotype variations. These differences could be due in part to the different genetic backgrounds in which the duplications arose, and in part to the often only approximate evaluation of the extent of the duplications. In fact, the only two patients with a molecular characterization of the breakpoint and analysed with an aCGH methodology were those reported by Peeters et al., [14] and Failla et al., [19].

We report a new case of a girl with a 8.5 Mb duplication of the 22q13.2-qter region translocated to 22p11. This region includes 120 genes, 54 of which expressed (normalized expression >1) in the brain (according to EBI Expression Atlas database [23]) and 9 almost exclusively expressed in brain. Due to the high number of genes, the genotype phenotype correlation is, therefore, extremely difficult. Inside this region maps the gene *SHANK3*. Recently, it has been reported that *SHANK3* dosage is critical for normal brain function and that its overexpression causes a hyperkinetic neuropsychiatric disorder characterized by manic-like behaviour and seizures consistent with synaptic excitatory/inhibitory imbalance [24]. Some of these characteristics have been observed, indeed, also in patients with the smallest 22q13.3-qter duplication [9, 19]. Although our patient displays a mild phenotype, comparable to that of patients with the smallest 22q13.3-qter duplication (Table 3), she does not present psychiatric signs. She presents slight mental retardation, microcephaly and minor dysmorphic traits such as telecanthus, large low set and laterally protruding ears. However, she does not display major malformation like cleft palate, genital or kidney malformations as those observed in samples with the 22q13.2-qter duplication [7, 9, 15, 17] (Table 3).

**Table 3:** Overview of clinical features of patients with a 22q13 to qter duplication

	Petek et al.	Biesecker et al.	Schinzel	Feenstra et al.	Our Patient	Feenstra et al.	Feenstra et al.	Okamoto et al.	Peeters et al.	Failla et al.
				(patient 1)		(patient 2)	(patient 3)	(patient 1)		
Duplicated region	q13-qter	q13.2-qter	q13.2-qter	q13.2-qter	q13.2-qter	q13.3-qter	q13.3-qter	q13.3-qter	q13.3-qter	q13.3-qter
Duplication cause	der(19)t(19p;22q)dn		der(6)t(6;22)mat	der(21)t(21p;22q)	der(22)t(22p;22q)dn	der(21)t(21p;22q)pat	der(21)t(21p;22q)dn	der(22)t(22p;22q)dn	der(22)t(22p;22q)dn	
Sex	M	F	F	F	F	M	M	F	F	F
Psychomotor retardation	+	+	+	+	mild	moderate	mild	moderate	mild	Moderate Disorganized schizophrenia
Stature (height)	−2.5 S.D.	0 S.D.	Short stature	−4 S.D.	0 S.D.	−3 S.D	−3 S.D	−3.2 SD	0 SD	
Hypotonia		+		+	−	−	−	+	−	+
*Head*										
Microcephaly	+	+	+	+	+	+	+	+	+	+
Brain abnormalities		+		−	−	−	−	−	−	−
Sparse, fine hair	+			+	−	+	+			
Prominent forehead				+	−	+	+	+	+	
Hypertelorism	−	+	+	+	+	−	+	+	−	+
Palpebral fissures slant up			+	+		−	−	−		
Narrow palpebral fissures				+		+	−	+	−	
Epicanthic folds	+			+	+	−	−			
Wide nasal bridge				+	+	−	−	+	+	
Low set ears	+	−	+	+	+	−	−	+	−	
Dysplastic ears		−		−	−	−	−	+	−	
Hearing loss		+	−	−	−	−	−	−	−	
Long philtrum				−	+	−	−			
Prominent upper lip				+	−	+	+		−	
Prominent lower lip				−	+	−	−		−	
Cleft lip		+	−	−	−					
Cleft palate		+	+	+	−	−	−	−	−	
*Thorax, abdomen and genitalia*										
Renal malformations	+			+	−	−	−	−	−	
Genital malformation	+			−	−	−	−	−	−	
*Limbs*										
Dysplastic hip				+	+	−	−			
Slender fingers					−					
Clinodactyly fifth finger				−	+					
Club foot, varus				−	−					
Survival	Alive, 9 years	Alive, 9 years	Alive, 8.5 years	Alive, 5 years	Alive, 15 years	Alive, 2 years	Alive, 40 years	Alive, 4 years	Alive, 6.8 years	Alive, 20 years

It is generally assumed that 22qter duplications originate from the missegregation of a familial balanced translocation or through an abnormal recombination of a parental chromosomal inversion [25, 26]. Pure duplication of distal 22q, however, is usually the consequence of a *de novo* non-reciprocal translocation of the distal part of chromosome 22 on the short arm of an acrocentric chromosome [9, 13, 14]. In this sense, the case reported here is not an exception, since the duplication observed is due to a *de novo* unbalanced translocation of the 22q13.2-qter region on the short arm of the paternal acrocentric chromosome 22.

In order to clarify the mechanism underlying the 22q rearrangement in our patient, we analysed the genotype of the SNPs in the 22q13.2-qter region both in the proband and her parents. Genotype analysis showed that the duplication involved the paternal chromosome 22 and that the two paternal copies of the 22q13.2-qter region had an identical haplotype. This demonstrates that both copies originated from the same paternal chromosome. Interestingly, also in the patient reported by Failla et al., [19] the duplication involved the paternal chromosome: the microsatellite analysis revealed the presence of one maternal and two paternal alleles. Since genotype information is not available for the other cases of 22qter pure trisomies, it is not possible to conclude whether this is a general mechanism or if it represents only one possible molecular event. To clarify this it would therefore be desirable to also characterise the oldest cases using SNP array.

The fact that, in our patient, the two paternal 22q13.2-qter copies have the same haplotype, enables us to rule out that the rearrangement is the result of a reciprocal translocation between the two paternal chromosomes 22 due to a non-allelic recombination during spermatogenesis.

Reciprocal translocations are the most common type of translocation and generally result from the swapping of chromosomal arms between heterologous chromosomes as a result of double strand breaks (DSBs) followed by non-allelic DNA repair mechanisms: non-homologous end join (NHEJ) or non-allelic homologous recombination (NAHR). Neither mechanism, however, is sufficiently adequate to describe the formation of non-reciprocal translocations, consequently other mechanisms need to be considered.

One model that could explain both the occurrence of a distal 22q intrachromosomal duplication and its non-reciprocal translocation on the short arm of an acrocentric chromosome, is a break-induced replication (BIR) which is involved in non-reciprocal translocation events in yeast [27–29]. BIR is a sub-pathway of the homologous recombination pathway used when only one DSB end shares homology with a template elsewhere in the genome (for example, in the case of telomere erosion).

**Fig. 5:**
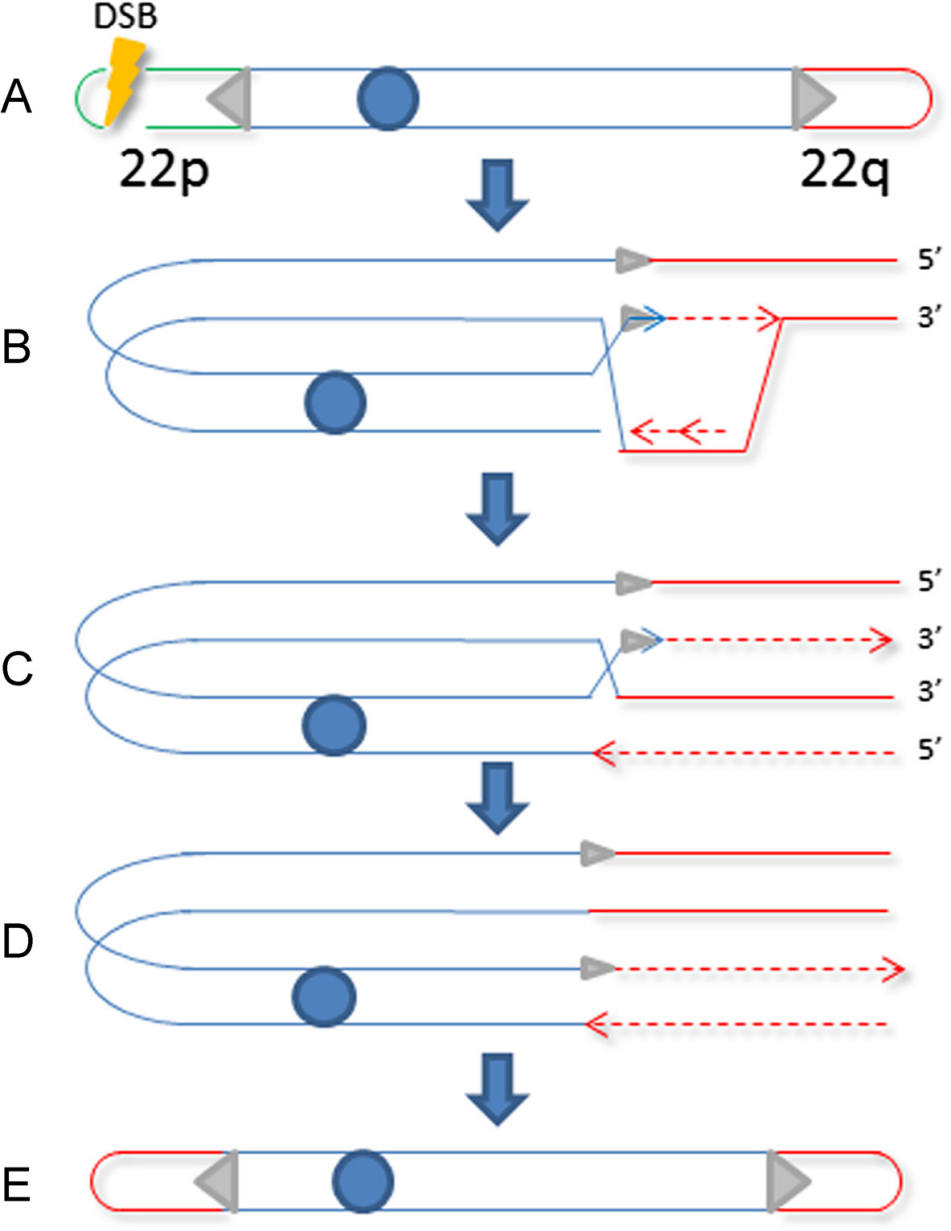
Mechanism proposed to explain the formation of a non-reciprocal translocation of the duplicated 22q13.2-qter region. The grey triangles are short regions of microhomology. In green and red are the distal portions of the 22p and 22q arms, respectively. The picture is not to scale. **a** A DSB eliminates the telomere of the short arm. **b** The 3′end proximal to the DSB invades a homolog sequence on the q arm and induces the formation of a mono-directional replication fork. The arrow indicates the direction of the replication fork. **c**-**d** The replication proceeds until the chromosome end is reached. **e** The distal portion of 22q (red) is duplicated and transposed inverted on 22p. See the discussion for details

In BIR, recombination is used to establish a uni-directional replication fork which can copy the template DNA till the end of the chromosome. If homologous sequences are located ectopically, BIR should result in the formation of a non-reciprocal translocation with loss of the telomeric part of the broken chromosome. It has been proposed that also regions of micro-homology are sufficient to induce a particular form of BIR defined micro-homologous-mediated BIR (MMBIR) [30]. Our hypothesis is that, on the distal part of the short arm of one chromosome 22, a DSB arose which resulted in the loss of the telomere. To stabilize the chromosome end, the 3’end of the 22p probably invaded a micro-homologous sequence located on the other chromosome arm, thus forming a unidirectional replication fork. The replication from this site presumably then produced a non-reciprocal translocation in which the 22q13.2-qter part was duplicated distal to the chromosome end produced by the DSB. Figure 5 shows the model proposed.

Clearly a prerequisite for MMBIR is the presence of short regions of homology at the breakpoint. Although the exact sequence of the breakpoint on the short arm of chromosome 22 is not known, however, it is known that this portion of the genome is rich in tandem repeats with a shared micro-homology with other genomic regions that might be the substrate for MMBIR. We are aware that what we are proposing is a hypothesis that only the sequencing of the breakpoints and the identification of regions of micro-homology can confirm or disprove.

## Conclusions

We have reported what we believe is a new case of pure duplication of 22q13.2, where we demonstrate that the combined use of FISH with both the copy number state and the genotype information, provided by SNP array, are not only important for the characterisation of the rearrangement and its origin, but also to shed light on the molecular mechanisms underlying cryptic chromosomal rearrangements.

## Consent

Fully informed parental consent was obtained for the publication of this case report together with Fig. 1. A copy of the written consent is available for review by the editor of this journal.

## Abbreviations

BAC: Bacterial artificial chromosome
BIR: Break-induced replication
DA-DAPI: Distamycin A and 4′,6-diamidino-2-phenylindole
DAPI: 4′,6-Diamidino-2-Phenylindole, Dilactate
DSBs: Double strand breaks
FISH: Fluorescence in situ hybridisation
MMBIR: Micro-homologous-mediated BIR
NAHR: Non-allelic homologous recombination
NHEJ: Non-homologous end join
NOR: Nucleolus organiser region
SNP: Single nucleotide polymorphism
WISCH: Wechsler intelligence scale for children

## References

[1] Gosden JR, Lawrie SS, Gosden CM (1981). Satellite DNA sequences in the human acrocentric chromosomes: information from translocations and heteromorphisms. Am J Hum Genet.

[2] Greig GM, Willard HF (1992). Beta satellite DNA: characterization and localization of two subfamilies from the distal and proximal short arms of the human acrocentric chromosomes. Genomics.

[3] Jones RS, Potter SS (1985). Characterization of cloned human alphoid satellite with an unusual monomeric construction: evidence for enrichment in HeLa small polydisperse circular DNA. Nucleic Acids Res.

[4] Ravnan JB, Tepperberg JH, Papenhausen P, Lamb AN, Hedrick J, Eash D, Ledbetter DH, Martin CL (2006). Subtelomere FISH analysis of 11 688 cases: an evaluation of the frequency and pattern of subtelomere rearrangements in individuals with developmental disabilities. J Med Genet.

[5] Abeliovich D, Maor E, Bashan N, Carmi R (1989). Duplication of distal 22q. Am J Med Genet.

[6] Barajas-Barajas LO, Valdez LL, Gonzalez JR, Garcia-Garcia C, Rivera H, Ramirez L (2004). Sensorineural deafness in two infants: a novel feature in the 22q distal duplication syndrome. Cardinal signs in trisomies 22 subtypes. Genet Couns.

[7] Biesecker LG, Rosenberg M, Dziadzio L, Ledbetter DH, Ning Y, Sarneso C, Rosenbaum K (1995). Detection of a subtle rearrangement of chromosome 22 using molecular techniques. Am J Med Genet.

[8] Cantu JM, Hernandez A, Vaca G, Plascencia L, Martinez-Basalo C, Ibarra B, Rivera H (1981). Trisomy 22q12 leads to qter: “aneusomie de recombinaison” of a pericentric inversion. Ann Genet.

[9] Feenstra I, Koolen DA, Van der Pas J, Hamel BC, Mieloo H, Smeets DF, Van Ravenswaaij CM (2006). Cryptic duplication of the distal segment of 22q due to a translocation (21;22): three case reports and a review of the literature. Eur J Med Genet.

[10] Fryns JP, De Backer D, Lemli L, Pedersen JC, Van den Berghe H (1980). Partial duplication of the long arm of chromosome 22 (22q 13) with complete 22 trisomy phenotype. Acta Paediatr Belg.

[11] Jensen PKA (1984). A 5p;22q reciprocal translocation with a high risk for segregation of unbalanced offspring. Clin Genet.

[12] Johnson MP, Greb A, Goyert G, Drugan A, Qureshi F, Sacks AJ, Evans MI (1990). Midtrimester diagnosis and anomalies in the dup(22q) syndrome: correlation of aneuploidy with low maternal serum alpha-fetoprotein and oligohydramnios. Am J Med Genet.

[13] Okamoto N, Kubota T, Nakamura Y, Murakami R, Nishikubo T, Tanaka I, Takahashi Y, Hayashi S, Imoto I, Inazawa J (2007). 22q13 Microduplication in two patients with common clinical manifestations: a recognizable syndrome?. Am J Med Genet A.

[14] Peeters H, Vermeesch J, Fryns JP (2008). A cryptic duplication 22q13.31 to qter leads to a distinct phenotype with mental retardation, microcephaly and mild facial dysmorphism. Genet Couns.

[15] Petek E, Kostl G, Mutz I, Wagner K, Kroisel PM (2000). Characterization of a de novo partial trisomy 22q13-qter in a patient by microFISH. Clin Dysmorphol.

[16] Rivera H, Garcia-Esquivel L, Romo MG, Perez-Garcia G, Martinez y Martinez R (1988). The 22q distal trisomy syndrome in a recombinant child. Ann Genet.

[17] Schinzel A, Niedrist D (2001). Chromosome imbalances associated with epilepsy. Am J Med Genet.

[18] Wieczorek D, Holtvogt J, Thonig S, Gillessen-Kaesbach G (1998). A female patient with partial duplication 22 (q13-->qter). Clin Dysmorphol.

[19] Failla P, Romano C, Alberti A, Vasta A, Buono S, Castiglia L, Luciano D, Di Benedetto D, Fichera M, Galesi O (2007). Schizophrenia in a patient with subtelomeric duplication of chromosome 22q. Clin Genet.

[20] Rooney DE (1992). Human cytogenetics a practical approach.

[21] ISCN (2013). ISCN 2013: An International System for Human Cytogenetic Nomenclature (2013).

[22] Piovani G, Borsani G, Bertini V, Kalscheuer VM, Viertel P, Bellotti D, Valseriati D, Barlati S (2006). Unexpected identification of two interstitial deletions in a patient with a pericentric inversion of a chromosome 4 and an abnormal phenotype. Eur J Med Genet.

[23] Petryszak R, Burdett T, Fiorelli B, Fonseca NA, Gonzalez-Porta M, Hastings E, Huber W, Jupp S, Keays M, Kryvych N (2014). Expression Atlas update--a database of gene and transcript expression from microarray- and sequencing-based functional genomics experiments. Nucleic Acids Res.

[24] Han K, Holder JL, Schaaf CP, Lu H, Chen H, Kang H, Tang J, Wu Z, Hao S, Cheung SW (2013). SHANK3 overexpression causes manic-like behaviour with unique pharmacogenetic properties. Nature.

[25] Mirza G, Imaizumi K, Ragoussis J (2000). Partial trisomy 22 in a liveborn resulting from a rearrangement between chromosomes 6 and 22. J Med Genet.

[26] Prasher VP, Roberts E, Norman A, Butler AC, Krishnan VH, McMullan DJ (1995). Partial trisomy 22 (q11.2-q13.1) as a result of duplication and pericentric inversion. J Med Genet.

[27] Bosco G, Haber JE (1998). Chromosome break-induced DNA replication leads to nonreciprocal translocations and telomere capture. Genetics.

[28] Haber JE (2006). Transpositions and translocations induced by site-specific double-strand breaks in budding yeast. DNA Repair (Amst).

[29] Myung K, Chen C, Kolodner RD (2001). Multiple pathways cooperate in the suppression of genome instability in Saccharomyces cerevisiae. Nature.

[30] Hastings PJ, Ira G, Lupski JR (2009). A microhomology-mediated break-induced replication model for the origin of human copy number variation. PLoS Genet.

